# A Skew Logistic Distribution for Modelling COVID-19 Waves and Its Evaluation Using the Empirical Survival Jensen–Shannon Divergence

**DOI:** 10.3390/e24050600

**Published:** 2022-04-25

**Authors:** Mark Levene

**Affiliations:** Department of Computer Science and Information Systems, Birkbeck, University of London, London WC1E 7HX, UK; mlevene@dcs.bbk.ac.uk

**Keywords:** empirical survival Jensen–Shannon divergence, Kolmogorov–Smirnov two-sample test, skew logistic distribution, bi-logistic growth, epidemic waves, COVID-19 data

## Abstract

A novel yet simple extension of the symmetric logistic distribution is proposed by introducing a skewness parameter. It is shown how the three parameters of the ensuing skew logistic distribution may be estimated using maximum likelihood. The skew logistic distribution is then extended to the skew bi-logistic distribution to allow the modelling of multiple waves in epidemic time series data. The proposed skew-logistic model is validated on COVID-19 data from the UK, and is evaluated for goodness-of-fit against the logistic and normal distributions using the recently formulated empirical survival Jensen–Shannon divergence (ESJS) and the Kolmogorov–Smirnov two-sample test statistic (KS2). We employ 95% bootstrap confidence intervals to assess the improvement in goodness-of-fit of the skew logistic distribution over the other distributions. The obtained confidence intervals for the ESJS are narrower than those for the KS2 on using this dataset, implying that the ESJS is more powerful than the KS2.

## 1. Introduction

In exponential growth, the population grows at a rate proportional to its current size. This is unrealistic, since in reality, growth will not exceed some maximum, called its carrying capacity. The logistic equation [1] (Chapter 6) deals with this problem by ensuring that the growth rate of the population decreases once the population reaches its carrying capacity [2]. Statistical modelling of the logistic equation’s growth and decay is accomplished with the *logistic distribution* [3] and [4] (Chapter 22), noting that the tails of the logistic distribution are heavier than those of the ubiquitous normal distribution. The normal and logistic distributions are both symmetric, however, real data often exhibits skewness [5], which has given rise to extensions of the normal distribution to accommodate for skewness, as in the skew normal [6] and epsilon skew normal [7] distributions. Subsequently, skew logistic distributions were also devised, as in [8,9].

Epidemics, such as COVID-19, are traditionally modelled by compartmental models such as the SIR (Susceptible-Infected-Removed) model and its extension, the SEIR (Susceptible-Exposed-Infected-Removed) model, which estimate the trajectory of an epidemic [10]. These models typically rely on assumptions on how the disease is transmitted and progresses [11], and are routinely used to understand the consequences of policies such as mask wearing and social distancing [12]. Time series models [13], on the other hand, employ historical data to make forecasts about the future, are generally simpler than compartmental models, and are able to make forecasts on, for example, number of cases, hospitalisations and deaths. The SIR model can be interpreted as a logistic growth model [14,15]. However, as the data is inherently skewed, a skewed logistic statistical model would be a natural choice, although, as such, it does not rely on biological assumptions in its forecasts [16].

Herein, we present a novel yet simple (one may argue the simplest), three parameter skewed extension to the logistic distribution to allow for asymmetry; c.f. [16]. Nevertheless, if instead of our extension we deploy one of the other skew logistic distributions (such as the one described in [8]) the results would no doubt be comparable to the results we obtain herein; however, we pursue our simpler extension, detailing its statistical properties.

In the context of analysing epidemics, the logistic distribution is normally preferred, as it is a natural distribution to use in modelling population growth and decay. However, we still briefly mention a comparison of the results we obtain in modelling COVID-19 waves with the skew logistic distribution, to one which, instead, employs a skew normal distribution (more specifically we choose the, flexible, epsilon skew normal distribution [7]). The result of this comparison implies that utilising the epsilon skew normal distribution leads, overall, to results which are comparable to those when utilising the skew logistic distribution. However, in practice, it is still preferable to make use of the skew logistic distribution as it is the natural model to deploy in this context [17], since, on the whole, it is more consistent with the data as its tails are heavier than those of a skew normal distribution.

Epidemics are said to come in “waves”. The precise definition of a wave is somewhat elusive [18], but it is generally accepted that, assuming we have a time series of the number of, say, daily hospitalisations, a wave will span over a period from one valley (minima) in the time series to another valley, with a peak (maxima) in between them. There is no strict requirement that waves do not overlap, although, for simplicity we will not consider any overlap as such; see [18], for an attempt to give an operational definition of the concept of epidemic wave. In order to combine waves, we make use of the concept of *bi-logistic growth* [19,20], or more generally, multi-logistic growth, which allows us to sum two or more instances of logistic growth when the time series spans over more than a single wave.

To fit the skew logistic distribution to the time series data we employ maximum likelihood, and to evaluate the goodness-of-fit we make use of the recently formulated *empirical survival Jensen–Shannon divergence* (ESJS) [21,22] and the well-established *Kolmogorov–Smirnov two-sample test statistic* (KS2) [23] (Section 6.3). The ESJS is an information-theoretic goodness-of-fit measure of a fitted parametric continuous distribution, which overcomes the inadequacy of the *coefficient of determination*, $R^{2}$, as a goodness-of-fit measure for nonlinear models [24]. The KS2 statistic also satisfies this criteria regarding $R^{2}$; however, we observe that the 95% bootstrap confidence intervals [25] we obtain for the ESJS are narrower than those for the KS2, suggesting that the ESJS is more powerful [26] than the KS2. Another well-known limitation of the KS2 statistic is that it is less sensitive to discrepancies at the tails of the distribution than the ESJS statistic is, in the sense that as opposed to ESJS it is “local”, i.e., its value is determined by a single point [27].

The rest of the paper is organised as follows. In Section 2, we introduce a skew logistic distribution, which is a simple extension of the standard, symmetric, logistic distribution obtained by adding to it a single skew parameter and derive some of its properties. In Section 3, we formulate the solution to the maximum likelihood estimation of the parameters of the skew logistic distribution. In Section 4, we make use of an extension of the skew logistic distribution to the bi-skew logistic distribution to model a time series of COVID-19 data items having more than a single wave. In Section 5, we provide analysis of daily COVID-19 deaths in the UK from 30 January 2020 to 30 July 2021, assuming the skew logistic distribution as an underlying model of the data. The evaluation of goodness-of-fit of the skew logistic distribution to the data makes use of the recently formulated ESJS, and compares the results to those when employing the KS2 instead. We observe that the same technique, which we applied to the analysis of COVID-19 deaths, can be used to model new cases and hospitalisations. Finally, in Section 6, we present our concluding remarks. It is worth noting that in the more general setting of information modelling, being able to detect epidemic waves may help supply chains in planning increased resistance to such adverse events [28]. We note that all computations were carried out using the Matlab software package.

## 2. A Skew Logistic Distribution

Here, we introduce a novel *skew logistic distribution*, which extends, in straightforward manner, the standard two parameter logistic distribution [3] and [4] (Chapter 22) by adding to it a skew parameter. The rationale for introducing the distribution is that, apart from its simple formulation, we believe that the maximum likelihood solution presented below is also simpler than those derived for other skew logistic distributions, such as the ones investigated in [8,9]. This point provides further justification for our skew logistic distribution when introducing the bi-skew logistic distribution in Section 4.

Now, let μ be a location parameter, *s* be a scale parameter and λ be a skew parameter, where s>0 and 0<λ<2. Then, the probability density function of the skew logistic distribution at a value *x* of the random variable *X*, denoted as f(x;λ,μ,s), is given by:

(1) $$f\left(x;\lambda ,\mu ,s\right)=\kappa_{\lambda }\frac{\exp\left(-\lambda \;\frac{x-\mu }{s}\right)}{s{\left(1+\exp\left(-\frac{x-\mu }{s}\right)\right)}^{2}},$$

noting that for clarity we write x−μ above as a shorthand for $\left(x-\mu \right)$, and $\kappa_{\lambda }$ is a normalisation constant, which depends on λ.

When λ=1, the skew logistic distribution reduces to the standard logistic distribution as in [3] and [4] (Chapter 22), which is symmetric. On the other hand, when 0<λ<1, the skew logistic distribution is positively skewed, and when 1<λ<2, it is negatively skewed, , and when 1<λ<2, it is negatively skewed. So, when λ=1, $\kappa_{\lambda }=1$, and, for example, when λ=0.5 or 1.5, $\kappa_{\lambda }=2/\pi$. For simplicity, from now on, unless necessary, we will omit to mention the constant $\kappa_{\lambda }$ as it will not effect any of the results.

The *skewness* of a random variable *X* [4,5], is defined as:

$$E\left[{\left(\frac{X-\mu }{s}\right)}^{3}\right],$$

and thus, assuming for simplicity of exposition (due the linearity of expectations [5]) that μ=0 and s=1, the skewness of the skew logistic distribution, denoted by γ(λ), is given by:

(2) $$\gamma \left(\lambda \right)=\int_{-\infty }^{\infty }x^{3}\;\frac{\exp\left(-\lambda x\right)}{s{\left(1+\exp\left(-x\right)\right)}^{2}}\;dx.$$

First, we will show that letting $\lambda_{1}=\lambda$, with $0<\lambda_{1}<1$, we have $\gamma (\lambda_{1})>0$, that is $f(x;\lambda_{1},0,1)$ is positively skewed. We can split the integral in (2) into two integrals for the negative part from −∞ to 0 and the positive part from 0 to ∞, noting that when x=0, the expression to the right of the integral is equal to 0. Then, on setting y=−x for the negative part, and y=x for the positive part, the result follows, as by algebraic manipulation it can be shown that:

(3) $$\frac{\exp(-\lambda_{1}y)}{{\left(1+\exp(-y)\right)}^{2}}>\frac{\exp(\lambda_{1}y)}{{\left(1+\exp(y)\right)}^{2}},$$

implying that $\gamma (\lambda_{1})>0$ as required.

Second, in a similar fashion to above, on letting $\lambda_{2}=\lambda_{1}+1=\lambda$, with $1<\lambda_{2}<2$, it follows that $\gamma (\lambda_{2})<0$, that is $f(x;\lambda_{2},0,1)$ is negatively skewed. In particular, by algebraic manipulation we have that:

(4) $$\frac{\exp\left(-\lambda_{2}y\right)}{{\left(1+\exp(-y)\right)}^{2}}<\frac{\exp\left(\lambda_{2}y\right)}{{\left(1+\exp(y)\right)}^{2}},$$

implying that $\gamma (\lambda_{2})<0$ as required.

The cumulative distribution function of the skew logistic distribution at a value *x* of the random variable *X* is obtained by integrating f(x;λ,μ,s), to obtain F(x;μ,s,λ), which is given by:

(5) $$\begin{array}{cc} F\left(x;\lambda ,\mu ,s\right)=\kappa_{\lambda }\exp\left(-\left(\lambda -2\right)\;\frac{x-\mu }{s}\right) & \left(\frac{1}{\left(1+\exp\left(\frac{x-\mu }{s}\right)\right)}\right.-\\ \; & \left.\;\;\frac{\lambda -1}{\lambda -2}\;{}_{2}F_{1}\left(1,2-\lambda ;3-\lambda ;-\exp\left(\frac{x-\mu }{s}\right)\right)\right), \end{array}$$

where ${}_{2}F_{1}\left(a,b;c;z\right)$ is the *Gauss hypergemoetric function* [29] (Chapter 15); we assume a,b and *c* are positive real numbers, and that *z* is a real number extended outside the unit disk by analytic continuation [30].

The hypergeometric function has the following integral representation [29] (Chapter 15),

(6) $$\frac{\Gamma (c)}{\Gamma (b)\Gamma (c-b)}\int_{0}^{1}\frac{t^{b-1}{\left(1-t\right)}^{c-b-1}}{{\left(1-tz\right)}^{a}}\;dt,$$

where c>b. Now, assuming without loss of generality that μ=0 and s=1, we have that:

(7) $${}_{2}F_{1}\left(1,2-\lambda ;3-\lambda ;-\exp(x)\right)=\left(2-\lambda \right)\int_{0}^{1}\frac{t^{1-\lambda }}{\left(1+t\;\exp(x)\right)}\;dt,$$

where *x* is a real number.

Therefore, from (7) it can be verified that: (i) ${}_{2}F_{1}\left(1,2-\lambda ;3-\lambda ;-\exp\left(x\right)\right)$ is monotonically decreasing with *x*, (ii) as *x* tends to plus infinity, ${}_{2}F_{1}\left(1,2-\lambda ;3-\lambda ;-\exp\left(x\right)\right)$ tends to 0 and (iii) as *x* tends to minus infinity, ${}_{2}F_{1}\left(1,2-\lambda ;3-\lambda ;-\exp\left(x\right)\right)$ tends to 1, since:

$$\left(2-\lambda \right)\int_{0}^{1}t^{1-\lambda }\;dt=1.$$

## 3. Maximum Likelihood Estimation for the Skew Logistic Distribution

We now formulate the maximum likelihood estimation [31] of the parameters μ,s and λ of the skew logistic distribution. Let $\left\{x_{1},x_{2},\ldots ,x_{n}\right\}$ be a random sample of *n* values from the density function of the skew logistic distribution in (1). Then, the log likelihood function of its three parameters is given by:

(8) $$\ln L\left(\lambda ,\mu ,s\right)=-n\ln\left(s\right)-\frac{\lambda }{s}\sum_{i=1}^{n}\left(x_{i}-\mu \right)-2\sum_{i=1}^{n}\ln\left(1+\exp\left(-\frac{x_{i}-\mu }{s}\right)\right).$$

In order to solve the log likelihood function, we first partially differentiate lnL(λ,μ,s) as follows:

(9) $$\begin{array}{cc} \frac{\partial \ln L(\lambda ,\mu ,s)}{\partial \lambda } & =\sum_{i=1}^{n}\frac{\mu -x_{i}}{s},\\ \frac{\partial \ln L(\lambda ,\mu ,s)}{\partial \mu } & =\frac{\lambda n}{s}-\frac{2}{s}\sum_{i=1}^{n}\frac{1}{1+\exp\left(\frac{x_{i}-\mu }{s}\right)}\;\mathrm{and}\\ \frac{\partial \ln L(\lambda ,\mu ,s)}{\partial s} & =-\frac{n}{s}+\frac{1}{s^{2}}\sum_{i=1}^{n}\left(x_{i}-\mu \right)\left(\lambda -\frac{2}{1+\exp\left(\frac{x_{i}-\mu }{s}\right)}\right). \end{array}$$

It is therefore implied that the maximum likelihood estimators are the solutions to the following three equations:

(10) $$\begin{array}{cc} \mu & =\frac{\sum_{i=1}^{n}x_{i}}{n},\\ \lambda & =\frac{2}{n}\sum_{i=1}^{n}\frac{1}{1+\exp\left(\frac{x_{i}-\mu }{s}\right)}\;\mathrm{and}\\ s & =\frac{1}{n}\sum_{i=1}^{n}\left(x_{i}-\mu \right)\left(\lambda -\frac{2}{1+\exp\left(\frac{x_{i}-\mu }{s}\right)}\right), \end{array}$$

which can be solved numerically.

We observe that the equation for μ in (10) does not contribute to solving the maximum likelihood, since the location parameter μ is equal to the mean only when λ=1. We thus look at an alternative equation for μ, which involves the mode of the skew logistic distribution.

To derive the mode of the skew logistic distribution we solve the equation,

(11) $$\frac{\partial }{\partial x}\frac{\exp\left(-\lambda \;\frac{x-\mu }{s}\right)}{s{\left(1+\exp\left(-\frac{x-\mu }{s}\right)\right)}^{2}}=0,$$

to obtain:

(12) $$\mu =x-s\;\log\left(-\frac{\lambda -2}{\lambda }\right).$$

Thus, motivated by (12) we replace the equation for μ in (10) with:

(13) $$\mu =m-s\;\log\left(-\frac{\lambda -2}{\lambda }\right),$$

where *m* is the mode of the random sample.

## 4. The Bi-Skew Logistic Distribution for Modelling Epidemic Waves

We start by defining the bi-skew logistic distribution, which will enable us to model more than one wave of infections at a time. We then discuss how we partition the data into single waves, in a way that we can apply the maximum likelihood from the previous section to the data in a consistent manner.

We present the *bi-skew logistic distribution*, which is described by the sum,

$$f\left(x;\lambda_{1},\mu_{1},s_{1}\right)+f\left(x;\lambda_{2},\mu_{2},s_{2}\right),$$

of two skew logistic distributions. It is given in full as:

(14) $$\frac{\exp\left(-\lambda_{1}\frac{x-\mu_{1}}{s_{1}}\right)}{s_{1}{\left(1+\exp\left(-\frac{x-\mu_{1}}{s_{1}}\right)\right)}^{2}}+\frac{\exp\left(-\lambda_{2}\frac{x-\mu_{2}}{s_{2}}\right)}{s_{2}{\left(1+\exp\left(-\frac{x-\mu_{2}}{s_{2}}\right)\right)}^{2}},$$

which characterises two distinct phases of logistic growth (c.f. [19,32]). We note that (14) can be readily extended to the general case of the sum of multiple skew logistic distributions; however, for simplicity, we only present the formula for the bi-skew logistic case. Thus, while the (single) skew logistic distribution can only model one wave of infected cases (or deaths, or hospitalisations), the bi-skew logistic distribution can model two waves of infections, and in the general cases, any number of waves.

In the presence of two waves, the maximum likelihood solution to (14), would give us access to the necessary model parameters, and solving the general case in the presence of multiple waves, when the sum in (14) may have two or more skew logistic distributions, is evidently even more challenging. Thus, we simplify the solution for the multiple wave case, and concentrate on an approximation assuming a sequential time series when one wave strictly follows the next. More specifically, we assume that each wave is modelled by a single skewed logistic distribution describing the growth phase until a peak is reached, followed by a decline phase; see [33] who consider epidemic waves in the context of the standard logistic distribution. Thus, a wave is represented by a temporal pattern of growth and decline, and the time series as a whole describes several waves as they evolve.

**Figure 1:**
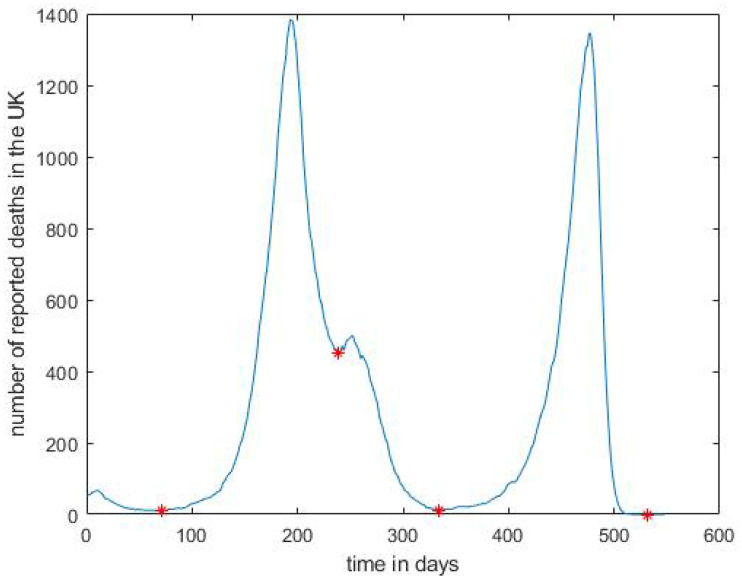
Reported daily COVID-19 deaths from 30 January 2020 to 30 July 2021 and their minima labelled ‘*’, resulting in four distinct waves; a moving average with a centred sliding window of 7 days was applied to the raw data.

To provide further clarification of the model, we mention that the skew-bi logistic distribution is *not* a *mixture model* per se, in which case there is a mixture weight for each distribution in the sum, as in, say, a Gaussian mixture [34] (Chapter 9). In the bi-skew logistic distribution case we do not have mixture weights, rather, we have two phases in our context waves, which are sequential in nature, possibly with some overlap, as can be seen in Figure 1 (c.f. [19,32]). Strictly speaking, the bi-skew logistic distribution can be viewed as a mixture model where the mixture weights are each 0.5 and a scaling factor of 2 is applied. Thus, as an approximation, we add a preprocessing step where we segment the time series into distinct waves, resulting in a considerable reduction to the complexity of the maximum likelihood estimation. We do, however, remark that the maximum likelihood estimation for the bi-skew logistic distribution is much simpler than that of a corresponding mixture model, due to the absence of mixture weights. In particular, although we could, in principle, make use of the EM (expectation-maximisation) algorithm [34] (Chapter 9) and [35] to approximate the maximum likelihood estimates of the parameters, this would not be strictly necessary in the bi-skew logistic case, cf. [36]. The only caveat, which holds independently of whether the EM algorithm is deployed or not, is the additional number of parameters present in the equations being solved. We leave this investigation as future work, and focus on our approximation, which does not require the solution to the maximum likelihood of (14); the details of the preprocessing heuristic we apply are given in the following section.

## 5. Data Analysis of COVID-19 Deaths in the UK

Here, we provide a full analysis of COVID-19 deaths in the UK from 30 January 2020 to 30 July 2021, employing the ESJS goodness-of-fit statistic and comparing it to the KS2 statistic. The daily UK COVID-19 data we used was obtained from [37].

As a proof of concept of the modelling capability of the skew logistic distribution, we now provide a detailed analysis of the time series of COVID-19 deaths in the UK from 30 January 2020 to 30 July 2021.

To separate the waves, we first smoothed the raw data using a moving average with a centred sliding window of 7 days. We then applied a simple heuristic, where we identified all the minima in the time series and defined a wave as a consecutive portion of the time, of at least 72 days, with the endpoints of each wave being local minima apart from the first wave, which starts from day 0. The resulting four waves in the time series are shown in Figure 1; see last column of Table 1 for the endpoints of the four waves. It would be worthwhile, as future work, to investigate other heuristics, which may, for example, allow overlap between the waves to obtain more accurate start and end points and to distribute the number of cases between the waves when there is overlap between them.

**Figure 2:**
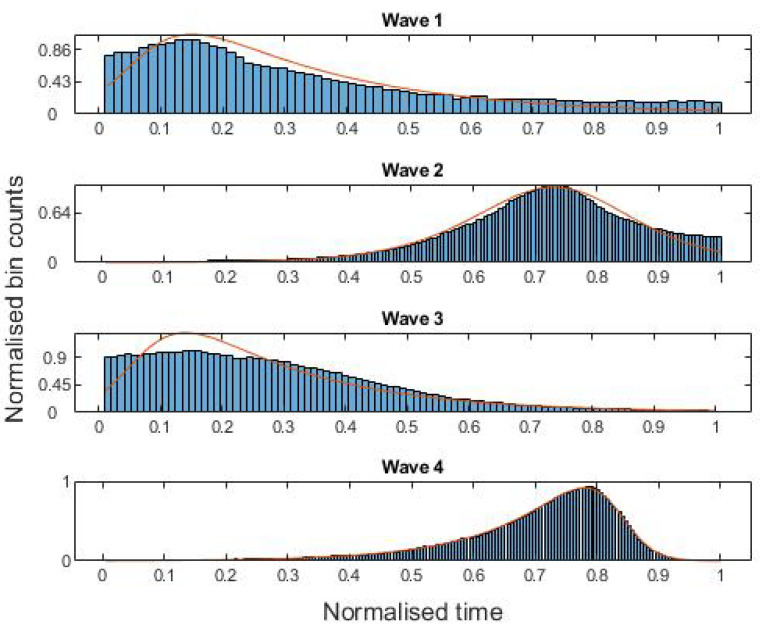
Histograms for the four waves of COVID-19 deaths from 30 January 2020 to 30 July 2021, each overlaid with the curve of the maximum likelihood fit of the skew logistic distribution to the data.

In Table 1, we show the parameters resulting from maximum likelihood fits of the skew logistic distribution to the four waves. Figure 2 shows histograms of the four COVID-19 waves, each overlaid with the curve of the maximum likelihood fit of the skew logistic distribution to the data. Pearson’s moment and median skewness coefficients [38] for the four waves are recorded in Table 2. It can be seen that the correlation between these and 1−λ is close to 1, as we would expect.

We now turn to the evaluation of goodness-of-fit using the ESJS (empirical survival Jensen–Shannon divergence) [21,22], which generalises the Jensen–Shannon divergence [39] to survival functions, and the well-known KS2 (Kolmogorov–Smirnov two-sample test statistic) [23] (Section 6.3). We will also employ 95% bootstrap confidence intervals [25] to measure the improvement in the ESJS and KS2, goodness-of-fit measures, of the skew-logistic over the logistic and normal distributions, respectively. For completeness, we formally define the ESJS and KS2.

To set the scene, we assume a time series [40], $x=\{x_{1},x_{2},\ldots ,x_{n}\}$, where $x_{t}$, for t=1,2,…,n is a value indexed by time, *t*, in our case modelling the number of daily COVID-19 deaths. We are, in particular, interested in the marginal distribution of x, which we suppose comes from an underlying parametric continuous distribution *D*.

The *empirical survival function* of a value *z* for the time series x, denoted by $\hat{S}\left(x\right)\left[z\right]$, is given by:

(15) $$\hat{S}\left(x\right)\left[z\right]=\frac{1}{n}\sum_{i=1}^{n}I_{\left\{x_{i}>z\right\}},$$

where *I* is the indicator function. In the following, we will let $\hat{P}\left(z\right)=\hat{S}\left(x\right)\left[z\right]$ stand for the empirical survival function $\hat{S}\left(x\right)\left[z\right]$, where the time series x is assumed to be understood from context. We will generally be interested in the empirical survival function $\hat{P}$, which we suppose arises from the survival function *P* of the parametric continuous distribution *D*, mentioned above.

The *empirical survival Jensen–Shannon divergence* (ESJS) between two empirical survival functions, ${\hat{Q}}_{1}$ and ${\hat{Q}}_{2}$ arising from the survival functions $Q_{1}$ and $Q_{2}$, is given by:

(16) $$ESJS\left({\hat{Q}}_{1},{\hat{Q}}_{2}\right)=\frac{1}{2}\;\int_{0}^{\infty }\;{\hat{Q}}_{1}\left(z\right)\;\log\left(\frac{{\hat{Q}}_{1}\left(z\right)}{\hat{M}(z}\right)\;+\;{\hat{Q}}_{2}\left(z\right)\;\log\left(\frac{{\hat{Q}}_{2}\left(z\right)}{\hat{M}\left(z\right)}\right)dz,$$

where:

$$\hat{M}\left(z\right)=\frac{1}{2}\;\left({\hat{Q}}_{1}\left(z\right)\;+\;{\hat{Q}}_{2}\left(z\right)\right).$$

We note that the ESJS is bounded and can thus be normalised, so it is natural to assume its values are between 0 and 1; in particular, when ${\hat{Q}}_{1}={\hat{Q}}_{2}$ its value is zero. Moreover, its square root is a metric [41], cf. [21].

The *Kolmogorov–Smirnov* two-sample test statistic between ${\hat{Q}}_{1}$ and ${\hat{Q}}_{2}$ as above, is given by:

(17) $$KS2\left({\hat{Q}}_{1},{\hat{Q}}_{2}\right)=\underset{z}{max}\left|{\hat{Q}}_{1}\left(z\right)-{\hat{Q}}_{2}\left(z\right)\right|,$$

where max is the maximum function, and |v| is the absolute value of a number *v*. We note that KS2 is bounded between 0 and 1, and is also a metric.

For a parametric continuous distribution *D*, we let $\phi =\phi (D,\hat{P})$ be the parameters that are obtained from fitting *D* to the empirical survival function, $\hat{P}$, using maximum likelihood estimation. In addition, we let $P_{\phi }=S_{\phi }\left(x\right)$ be the survival function of x, for *D* with parameters ϕ. Thus, the empirical survival Jensen–Shannon divergence and the Kolmogorov–Smirnov two-sample test statistic, between $\hat{P}$ and $P_{\phi }$, are given by $ESJS(\hat{P},P_{\phi })$ and $KS2(\hat{P},P_{\phi })$, respectively, where $\hat{P}$ and $P_{\phi }$ are omitted below as they will be understood from context. These values provide us with two measures of goodness-of-fit for how well *D* with parameters ϕ is fitted to x [22].

We are now ready to present the results of the evaluation. In Table 3, we show the ESJS values for the four waves and the said improvements, while in Table 4, we show the corresponding KS2 values and improvements. In all cases, the skew logistic is a preferred model over both the logistic and normal distributions, justifying the addition of a skewness parameter as can be see in Figure 2. Moreover, in all but one case the logistic distribution was preferred over the normal distribution—wave 3, where the KS2 statistic of the normal distribution was smaller than that of the logistic distribution. We observe that, for the second wave, the ESJS and KS2 values for the skew logistic and logistic distribution were the closest, since, as can be seen from Table 1, the second wave was more or less symmetric, in which case the skew logistic distribution reduces to the logistic distribution.

In Table 5 and Table 6, we present the bootstrap 95% confidence intervals of the ESJS and KS2 improvements, respectively, using the *percentile* method, while in Table 7 and Table 8, we provide the 95% confidence intervals of the ESJS and KS2 improvements, respectively, using the *bias-corrected and accelerated* (BCa) method [25], which adjusts the confidence intervals for bias and skewness in the empirical bootstrap distribution. In all cases, the mean of the bootstrap samples is above zero with a very tight standard deviation. As noted above, the second wave is more or less symmetric, so we expect that the standard logistic distribution will provide a fit to the data, which is as good as the skew logistic fit. It is thus not surprising that in this case the improvement percentages are, generally, not significant. In addition, the improvements for the third wave are also, generally, not significant, which may be due to the starting point of the third wave, given our heuristic, being close to its peak; see Figure 1. We observe that, for this dataset, it is not clear whether deploying the BCa method yields a significant advantage over simply deploying the percentile method.

In Table 9, we show the mean and standard deviation statistics of the confidence interval widths, of the metrics we used to compare the distributions, implying that, in general, the ESJS goodness-of-fit measure is more powerful than the KS2 goodness-of-fit measure. This is based on the known result that statistical tests using measures resulting in smaller confidence intervals are normally considered to be more powerful, implying that a smaller sample size may be deployed [42].

As mentioned in the introduction, we obtained comparable results to the above when modelling epidemic waves with the epsilon skew normal distribution [7] as opposed to using the skew logistic distribution; see also [43] for a comparison of a skew logistic and skew normal distribution in the context of insurance loss data, showing that the skew logistic performed better than the skew normal distribution for fitting the datasets tested. Further to the note in the introduction that the skew logistic distribution is a more natural one to deploy in this case due to its heavier tails, we observe that in an epidemic scenario, the number of cases counted can only be non-negative, while the epsilon skew normal also supports negative values.

## 6. Concluding Remarks

We have proposed the skew-logistic and bi-logistic distributions as models for single and multiple epidemic waves, respectively. The model is a simple extension of the symmetric logistic distribution, which can readily be deployed in the presence of skewed data that exhibits growth and decay. We provided validation for the proposed model using the ESJS as a goodness-of-fit statistic, showing that it is a good fit to COVID-19 data in UK and more powerful than the alternative KS2 statistic. As future work, we could use the model to compare the progression of multiple waves across different countries, extending the work of [16].

## Figures and Tables

**Table 1:** Parameters from maximum likelihood fits of the skew logistic distribution to the four waves, and the day of the local minimum (End), which is the end point of the wave.

Fitted Parameters for the Skew Logistic Distribution				
**Wave**	λ	μ	s	**End**
1	0.2150	3.5137	3.8443	71
2	1.0741	196.5157	14.4323	239
3	0.2297	243.0709	4.5882	334
4	1.7306	502.2758	7.0195	532

**Table 2:** Pearson’s moment and median skewness coefficients for the four waves, and the correlation between 1−λ and these coefficients.

Skewness			
**Wave**	1−λ	**Moment**	**Median**
1	0.7850	0.9314	0.2939
2	−0.0741	−0.7758	−0.0797
3	0.7703	0.9265	0.1939
4	−0.7306	−1.5555	−0.2413
Correlation		0.9931	0.9826

**Table 3:** ESJS values for the skew logistic (SL), logistic (Logit) and normal (Norm) distributions, and the improvement percentage of the skew logistic over the logistic (SL-Logit) and normal (SL-Norm) distributions, respectively.

ESJS Values for SL, Logit and Norm Distributions					
**Wave**	**SL**	**Logit**	**SL-Logit**	**Norm**	**SL-Norm**
1	0.0419	0.0583	28.25%	0.0649	35.54%
2	0.0392	0.0448	12.52%	0.0613	36.17%
3	0.0316	0.0387	18.38%	0.0423	25.38%
4	0.0237	0.0927	74.47%	0.0939	74.79%

**Table 4:** KS2 values for the skew logistic (SL), logistic (Logit) and normal (Norm) distributions, and the improvement percentage of the skew logistic over the logistic (SL-Logit) and normal (SL-Norm) distributions, respectively.

KS2 Values for SL, Logit and Norm Distributions					
**Wave**	**SL**	**Logit**	**SL-Logit**	**Norm**	**SL-Norm**
1	0.0621	0.1245	50.14%	0.1280	51.50%
2	0.0357	0.0391	8.57%	0.0420	15.01%
3	0.0571	0.0930	38.66%	0.0854	33.18%
4	0.0098	0.0817	87.98%	0.1046	90.61%

**Table 5:** Results from the percentile method for the confidence interval of the difference of the ESJS between the logistic (Logit) and skew logistic (SL), and between the normal (Norm) and skew logistic (SL) distributions, respectively; Diff, LB, UB, CI, Mean and STD stand for difference, lower bound, upper bound, confidence interval, mean of samples and standard deviation of samples, respectively.

Percentile Confidence Intervals for ESJS Improvement					
**Wave/Diff**	**LB of CI**	**UB of CI**	**Width of CI**	**Mean**	**STD**
1/SL-Logit	0.0093	0.0317	0.0224	0.0211	0.0063
1/SL-Norm	0.0170	0.0382	0.0212	0.0278	0.0063
2/SL-Logit	*−0.0010*	0.0066	0.0076	0.0034	0.0049
2/SL-Norm	0.0154	0.0232	0.0078	0.0201	0.0051
3/SL-Logit	*−0.0028*	0.0112	0.0140	0.0083	0.0022
3/SL-Norm	0.0021	0.0149	0.0128	0.0120	0.0022
4/SL-Logit	0.0549	0.0810	0.0261	0.0714	0.0068
4/SL-Norm	0.0560	0.0821	0.0261	0.0722	0.0070

**Table 6:** Results from the percentile method for the confidence interval of the difference of the KS2 between the logistic (Logit) and skew logistic (SL), and between the normal (Norm) and skew logistic (SL) distributions, respectively; Diff, LB, UB, CI, Mean and STD stand for difference, lower bound, upper bound, confidence interval, mean of samples and standard deviation of samples, respectively.

Percentile Confidence Intervals for KS2 Improvement					
**Wave/Diff**	**LB of CI**	**UB of CI**	**Width of CI**	**Mean**	**STD**
1/SL-Logit	0.0438	0.0760	0.0322	0.0621	0.0073
1/SL-Norm	0.0411	0.0821	0.0410	0.0684	0.0078
2/SL-Logit	0.0003	0.0047	0.0044	0.0033	0.0009
2/SL-Norm	0.0007	0.0092	0.0085	0.0065	0.0017
3/SL-Logit	*−0.0073*	0.0441	0.0514	0.0343	0.0082
3/SL-Norm	*−0.0142*	0.0365	0.0507	0.0267	0.0080
4/SL-Logit	0.0474	0.0728	0.0254	0.0680	0.0046
4/SL-Norm	0.0710	0.0962	0.0252	0.0905	0.0048

**Table 7:** Results from the BCa method for the confidence interval of the difference of the ESJS between the logistic (Logit) and skew logistic (SL), and between the normal (Norm) and skew logistic (SL) distributions, respectively; Diff, LB, UB, CI, Mean and STD stand for difference, lower bound, upper bound, confidence interval, mean of samples and standard deviation of samples, respectively.

BCa Confidence Intervals for ESJS Improvement					
**Wave/Diff**	**LB of CI**	**UB of CI**	**Width of CI**	**Mean**	**STD**
1/SL-Logit	0.0087	0.0260	0.0173	0.0210	0.0062
1/SL-Norm	0.0165	0.0333	0.0168	0.0275	0.0063
2/SL-Logit	*−0.0009*	0.0258	0.0267	0.0036	0.0053
2/SL-Norm	0.0153	0.0425	0.0272	0.0201	0.0050
3/SL-Logit	*−0.0024*	0.0095	0.0119	0.0084	0.0023
3/SL-Norm	*−0.0027*	0.0135	0.0162	0.0119	0.0024
4/SL-Logit	0.0308	0.0703	0.0395	0.0708	0.0074
4/SL-Norm	0.0554	0.0713	0.0159	0.0726	0.0069

**Table 8:** Results from the BCa method for the confidence interval of the difference of the KS2 between the logistic (Logit) and skew logistic (SL), and between the normal (Norm) and skew logistic (SL) distributions, respectively; Diff, LB, UB, CI, Mean and STD stand for difference, lower bound, upper bound, confidence interval, mean of samples and standard deviation of samples, respectively.

BCa Confidence Intervals for KS2 Improvement					
**Wave/Diff**	**LB of CI**	**UB of CI**	**Width of CI**	**Mean**	**STD**
1/SL-Logit	0.0428	0.0801	0.0373	0.0624	0.0074
1/SL-Norm	0.0444	0.0777	0.0333	0.0683	0.0078
2/SL-Logit	0.0005	0.0047	0.0042	0.0033	0.0008
2/SL-Norm	0.0001	0.0089	0.0088	0.0064	0.0017
3/SL-Logit	0.0013	0.0445	0.0432	0.0346	0.0077
3/SL-Norm	*−0.0111*	0.0368	0.0479	0.0263	0.0082
4/SL-Logit	0.0491	0.0739	0.0248	0.0676	0.0047
4/SL-Norm	0.0685	0.0985	0.0300	0.0908	0.0046

**Table 9:** Mean and standard deviation (STD) statistics for the confidence interval (CI) widths using the percentile (P) and BCa methods.

Summary Statistics for the CI Widths				
**Statistic**	ESJS **-P**	KS2 **-P**	ESJS **-BCa**	KS2 **-BCa**
Mean	0.0172	0.0298	0.0214	0.0287
STD	0.0077	0.0176	0.0091	0.0155

## Data Availability

Publicly available datasets were analysed in this study. This data can be found here: https://coronavirus.data.gov.uk/details/download, accessed on 20 March 2022.
